# The role of neck ultrasound in the follow-up of low- and intermediate- risk papillary thyroid cancer

**DOI:** 10.20945/2359-3997000000485

**Published:** 2022-06-27

**Authors:** Sara Gomes de Campos Lopes, David Nuno Dias Silva Ferreira, Vera Adriana Ribeiro Fernandes, Helena Manuela da Costa Cardoso Marques, Ricardo Fernando da Silva Santos Pereira, Ana Margarida Carvalho Monteiro

**Affiliations:** 1 Hospital de Braga Departamento de Endocrinologia Braga Portugal Departamento de Endocrinologia, Hospital de Braga, Braga, Portugal; 2 Universidade do Minho Escola de Medicina Braga Portugal Escola de Medicina, Universidade do Minho, Braga, Portugal; 3 Hospital de Braga Departamento de Cirurgia Geral Braga Portugal Departamento de Cirurgia Geral, Hospital de Braga, Braga, Portugal

## Abstract

**Objective:**

The optimal time for a neck ultrasound (US) in the follow-up of papillary thyroid cancer (PTC) after the first year is undetermined. We aimed to verify the utility of routine neck US in the surveillance of patients diagnosed with low- and intermediate-risk PTC with no evidence of disease at the one-year assessment.

**Materials and methods:**

We conducted a retrospective longitudinal study of patients with low- and intermediate-risk PTC with normal neck US, unstimulated serum thyroglobulin (Tg) < 1 ng/mL and negative anti-Tg antibodies at the one-year follow-up. Patients were divided into group 1 [undetectable Tg (<0.20 ng/mL)] and group 2 [detectable Tg but < 1 ng/mL]. The negative predictive value (NPV) of the one-year unstimulated Tg at the five-year and last follow-up visits was calculated.

**Results:**

We included n = 88 patients in group 1 and n = 8 patients in group 2. No patient from group 1 presented suspicious US findings at the five-year evaluation [NPV: 100.0% (95% confidence interval (CI): 95.5%-100.0%)], and at the last visit, only one patient had developed a lymph node classified as suspicious [NPV: 98.8% (95% CI: 93.2%-100.0%); mean follow-up: 6.7 years]. In group 2, two patients’ USs presented suspicious findings at the five-year evaluation [NPV: 75.0% (95% CI: 34.9%-96.8%)]. At the last visit, only one patient persisted with suspicious findings in the US [NPV: 87.5% (95% CI: 47.4%-99.7%); mean follow-up: 6.5 years].

**Conclusion:**

Low- and intermediate-risk PTC with an excellent response to treatment at the one-year assessment can be safely monitored with regular unstimulated Tg assessments. Conclusions should not be drawn for Tg levels between 0.20-0.99 ng/mL.

## INTRODUCTION

Differentiated thyroid cancer (DTC) represents the most common form of thyroid cancer, and the vast majority of these malignancies exhibit low or intermediate risk of recurrence ( 1 - 5 ). Consequently, DTC is associated with a favorable prognosis and very low mortality rates ( 1 , 3 ). Papillary thyroid cancer (PTC) is the most frequent form of DTC, accounting for 75%-85% of all thyroid cancers ( 6 , 7 ). According to the 2015 American Thyroid Association (ATA) guidelines ( 1 ), the management of patients with DTC, including PTC, is based on the monitoring of unstimulated serum thyroglobulin (Tg), anti-Tg antibodies (TgAb) and thyroid-stimulating hormone (TSH) levels and through neck ultrasound (US), according to the individual’s risk of recurrence (low, intermediate or high). Initially, it is advisable to measure the Tg and TgAb levels every 6 to 12 months and, subsequently, if the patient shows an excellent response to therapy, every 12 to 24 months. A neck US 6-12 months after the thyroidectomy and periodically thereafter is also recommended, based on the risk of recurrence and levels of Tg. The follow-up of patients for whom the one-year US is negative and serum Tg levels are low (<0.20 ng/mL or <1 ng/mL after TSH stimulation) can primarily be completed only with unstimulated Tg measurements. Nevertheless, the timing for repeating the US is not clarified, possibly leading to frequent and unnecessary exams.

Some authors consider neck US the main assessment tool in the surveillance of DTC due to its high resolution, easy access, non-invasiveness, speed of execution and usefulness in the qualitative characterization of the thyroid parenchyma and respective lymph nodes as well as the detection of recurrence ( 8 - 10 ). Conversely, some studies have shown insufficient evidence to support this follow-up procedure and that the early detection of recurrence may lead to unnecessary medical interventions without improvements in quality of life or life expectancy ( 7 , 11 ).

Recent studies have also shown that the increasing use of neck USs has led to more therapeutic interventions, such as surgeries and radioiodine remnant ablation (RRA), but not with an increase in survival rates ( 4 , 11 ). Moreover, a neck US only detects local metastasis, is an operator-dependent procedure and is associated with high false-positive rates. Three recently conducted studies reported false-positives in 67% ( 12 ), 57% ( 13 ) and 43.9% ( 14 ) of the patients, but only 1.2% ( 12 ) to 10% ( 13 ) of patients had a true recurrence. Similarly, two other studies have shown that a routine neck US for patients with reduced risk of recurrence has a very low probability of identifying recurrence ( 15 , 16 ), even if initially suspicious findings were detected ( 16 ).

Increasing evidence supports the performance of clinical assessment, neck US and measurement of unstimulated serum Tg levels at the one-year evaluation of low- and intermediate-risk DTC and, in subsequent years, only a neck US for patients in whom levels of Tg exceed the threshold of 1 ng/mL ( 14 , 17 , 18 ). Other investigators recommend a neck US be performed one to two years after the thyroidectomy and, over the next five years, only to perform one or two neck USs ( 19 ).

The aim of this study was to assess the utility of routine neck USs in the surveillance of patients diagnosed with low- and intermediate-risk PTC ( 1 ) with negative US findings, unstimulated undetectable or indeterminate serum Tg levels (<0.20 ng/mL and 0.21 to 0.99 ng/mL, respectively) and negative TgAb titers at their one-year follow-up visit (FUV-1).

## MATERIALS AND METHODS

### Type of study

This was a retrospective longitudinal single-center study conducted at the Endocrinology Department of the Hospital de Braga.

### Ethical considerations

The study was approved by the Ethics Committee for Health of the Hospital de Braga and the Ethics Committee for Life and Health Sciences of the University of Minho. For the entire duration of the study, we ensured the patients’ anonymity and confidentiality.

### Patients’ selection

We reviewed data from patients followed in the Thyroid Group Consultation (TGC) between January 2017 and October 2020 with low- and intermediate-risk PTC, according to the 2015 ATA guidelines ( 1 ). The case records were collected from the center’s information system database, Glintt^®^ (Heathcare Solutions, Portugal).

The inclusion criteria were the following: i) age at diagnosis of 18 years old or higher; ii) histologically confirmed diagnosis of PTC; iii) risk of recurrence classified as low or intermediate, according to the ATA Initial Risk Stratification System ( 1 ); iv) primary treatment consisting of total thyroidectomy (with or without central and/or lateral neck dissection and with or without RRA); v) Tg, TgAb and TSH levels at FUV-1 and at the five-year follow-up visit (FUV-5) available; and vi) neck US reports of FUV-1 and FUV-5.

The exclusion criteria were i) patients with suspicion or evidence of disease and/or TgAb positivity at FUV-1, ii) histological diagnosis of other DTC: follicular or Hürthle cell carcinoma or aggressive DTC variants (e.g., hobnail, tall-cell, columnar cell), and iii) any missing data from the inclusion criteria.

All the patients who met the above criteria were included in the study.

### Study design

The selected patients underwent thyroidectomy between 2011 and 2015. Our general surgery department utilized an institutional protocol based on recommendations the American Thyroid Association published in 2009. When the histopathology report confirmed a diagnosis of PTC, the patients were referred to the TGC. The TGC follow-up protocol for PTC consists of an initial assessment in the first 12 months after surgery and subsequent reassessments at least once a year. The follow-up includes

- Determination of serum Tg levels (IMMULITE^®^ 2000 Thyroglobulin, Siemens Healthcare Diagnostics Inc, functional sensitivity 0.9 ng/mL, analytical sensitivity 0.2 ng/mL);
- Determination of serum TSH levels [IMMULITE^®^, Siemens Healthcare Diagnostics Inc (until 2008); Cobas^®^ Elecsys TSH, Roche Diagnostics GmbH (from 2008 to 2010); Dimension Vista^®^ 1500 System, Siemens Healthcare Diagnostics Inc (from 2011 to 2020); Atellica^TM^ IM Thyroid Stimulating Hormone (TSH) assay, Siemens Healthcare Diagnostics Inc (since 2020)];
- Determination of serum TgAb levels [IMMULITE^®^ 2000 Anti-Tg Ab, Siemens Healthcare Diagnostics Inc (until July 2019); Atellica^TM^ IM Anti-Thyroglobulin (aTG), Siemens Healthcare Diagnostics Inc (since July 2019)]; and
- High-resolution gray-scale and color Doppler US studies of the thyroid bed and neck lymph node compartments.

Additional procedures are requested in accordance with the existing evidence-based guidelines ( 1 ).

For the purpose of the study, we collected and reviewed the laboratory data (unstimulated serum Tg, TgAb and TSH levels) and neck US from FUV-1, FUV-5 and the last available follow-up visit (last FUV).

Unstimulated serum Tg levels were classified as follows:

- Negative (Tg-N) if undetectable, that is, <0.20 ng/mL;
- Indeterminate (Tg-I) if detectable but <1 ng/mL;
- Suspicious (Tg-S) if ≥1 ng/mL.

Based on the classification the European Thyroid Association proposed in 2013 ( 9 ), we classified each node seen in the neck US as:

- normal (preserved hilum, ovoid shape, normal size, absent or hilar vascularization, and no other suspicious sign),
- indeterminate (absent hilum and one or more of the following: round shape, increased short axis, or increased central vascularization), or
- suspicious (at least one of the following: microcalcifications, partially cystic appearance, peripheral or diffusely increased vascularization, thyroid-like hyperechoic tissue).

At each time point and based on this classification ( 9 ), we classified the patients’ neck US as US-N if all the nodes were considered normal, US-I if no suspicious nodes but one or more indeterminate node was present or US-S if at least one node was classified as suspicious. The investigators reviewed and classified each neck US.

Furthermore, at each FUV, patients’ therapy response was evaluated according to the ATA guidelines ( 1 ) as follows: excellent response if no clinical, biochemical or structural evidence of disease is present; biochemical incomplete response if, in the absence of localizable disease, abnormal Tg or rising TgAb levels are detected; structural incomplete response if persistent or newly identified loco-regional or distant metastases are present; indeterminate response if nonspecific biochemical or structural findings that cannot be confidently classified as either benign or malignant are present, including patients with stable or declining TgAb levels without definitive structural evidence of disease.

The demographic characteristics (age and sex), follow-up time, execution of neck dissection and RRA and tumor histologic characteristics (variant, size, foci, extrathyroidal extension and lymphovascular invasion) were also collected.

### Data analysis

All the data was collected using Microsoft Office Excel^®^, and the analysis was conducted with IBM^®^ Statistical Package for the Social Sciences (IBM^®^ SPSS^®^ Statistics, version 26.0).

**Figure 1:**
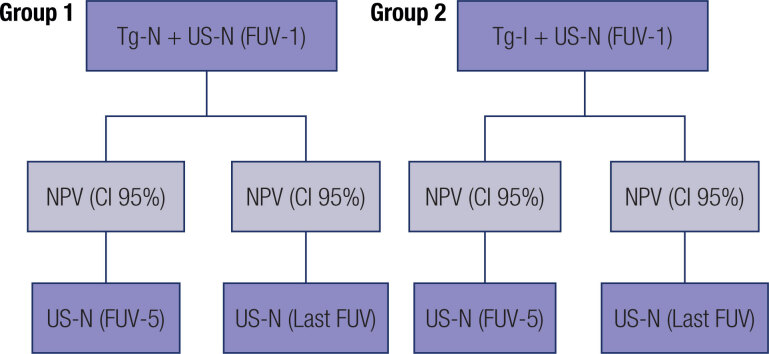
Study design flowchart. CI: confidence interval; FUV: follow-up visit; NPV: negative predictive value; Tg-I: indeterminate thyroglobulin levels; Tg-N: negative thyroglobulin levels; US-N: normal ultrasound.

Patients were divided into two groups, according to their FUV-1 unstimulated serum Tg levels: group 1 with undetectable levels (<0.2 ng/mL) and group 2 with indeterminate (0.20-0.99 ng/mL) ( Figure 1 ). To assess the normality of the distribution of the continuous variables in each group, we conducted the Shapiro-Wilk test. If normality was assured, values are presented in mean ± standard deviation. If normality was not assured, values are presented in median and interquartile range (IQR). Categorical variables are presented as absolute value (n) and relative frequency (%).

We calculated the negative predictive value (NPV) of the FUV-1 unstimulated serum Tg for group 1 and group 2. For this analysis, we adopted two definitions of test negativity: i) an undetectable level (<0.20 ng/mL) and ii) and indeterminate level (0.20-0.99 ng/mL) of serum Tg. We evaluated its ability to predict the absence of disease, defined as neck lymph node status of US-N, at two time points: i) FUV-5 and ii) the last FUV available.

Subsequently, as suspicious Tg levels may also indicate occult persistent disease, we also applied a composite endpoint to define absence of disease (both serum Tg < 1 ng/mL and US-N status) at the last FUV. We used a confidence interval of 95% to calculate all NPVs.

## RESULTS

We included a total of 184 adult patients diagnosed with PTC and submitted to total thyroidectomy, with follow-up in the TGC. After excluding 68 patients for missing data, six for suspicious Tg levels (Tg-S; >1 ng/mL), 10 for TgAb positivity at FUV-1 and 12 for neck lymph node status of US-I or US-S at FUV-1, we included a total of 88 cases.

A total of 80 cases had serum Tg levels<0.2 ng/mL (Tg-N) – group 1 – and 8 between 0.20 and 0.99 ng/mL (Tg-I) – group 2. We analyzed these two groups separately and determined their FUV-1 serum Tg status NPV. Table 1 shows our population’s baseline characteristics.

**Table 1:** Baseline characteristics of group 1 and group 2

		Group 1 (n = 80)	Group 2 (n = 8)
Age (years)		48.3 ± 13.6	46.9 ± 16.4
Gender			
	Female	67 (83.8%)	8 (100.0%)
	Male	13 (16.3%)	0 (0%)
Follow-up time (years)		6.7 ± 1.2	6.5 ± 1.8
	Neck dissection		
	Not done	33 (41.3%)	2 (25.0%)
	Central compartment dissection	41 (51.3%)	6 (75.0%)
	Central and lateral compartment dissection	6 (7.5%)	0 (0%)
	Lateral compartment dissection	0 (0%)	0 (0%)
Tumor size (cm)		1.2 (0.8-1.7)	1.2 (0.7-2.7)
Tumor foci			
	Unifocal	61 (76.3%)	6 (75.0%)
	Multifocal	19 (23.8%)	2 (25.0%)
Extrathyroidal extension			
	None	51 (63.8%)	6 (75.0%)
	Microscopic	29 (36.3%)	2 (25.0%)
Lymphovascular invasion			
	None	57 (71.3%)	6 (75.0%)
	Lymphatic invasion	13 (16.3%)	2 (25.0%)
	Vascular invasion	6 (7.5%)	0 (0%)
	Lymphatic and vascular invasion	4 (5.0%)	0 (0%)
Radioiodine remnant ablation			
	No	10 (12.5%)	2 (25.0%)
	Yes	70 (87.5%)	6 (75.0%)
Serum Tg and neck lymph node status		Tg-N + US-N	Tg-I + US-N
Estimated risk of recurrence a			
	Low	33 (41.3%)	6 (75.0%)
	Intermediate	43 (53.8%)	2 (25.0%)

Variables presented in mean ± standard deviation, median (IQR) or frequency n (%), with decimal approximation.

a According to the 2015 ATA guidelines ( 1 ).

### Group 1

At FUV-5, all the patients had a neck lymph node status classified as US-N [NPV of the Tg-N + US-N status at FUV-1: 100% (95% CI: 95.5% to 100.0%)]. Thirteen patients presented with a Tg-I status and three patients with a Tg-S status ( Table 2 ).

**Table 2:** Group 1 (n = 80) and group 2 (n = 8): unstimulated serum Tg and neck lymph node status at FUV-5 and at last FUV

		Group 1				Group 2			
		Neck Lymph Node Status at FUV-5				Neck Lymph Node Status at FUV-5			
		US-N (%)	US-I (%)	US-S (%)	Total (%)	US-N (%)	US-I (%)	US-S (%)	Total (%)
Serum Tg	Tg-N, <0.2 ng/mL	64 (80.0)	0 (0)	0 (0)	64 (80.0)	3 (37.5)	1 a , b (12.5)	0 (0)	4 (50.0)
status	Tg-I, 0.2-0.99 ng/mL	13 (16.3)	0 (0)	0 (0)	13 (16.3)	3 (37.5)	0 (0)	1 a , c , d (12.5)	4 (50.0)
at FUV-5	Tg-S, ≥1 ng/mL	3a (3.8)	0 (0)	0 (0)	3 (3.8)	0 (0)	0 (0)	0 (0)	0 (0)
Total		80 (100)	0 (0)	0 (0)	80 (100)	6 (75.0)	1 (12.5)	1 (12.5)	8 (100)
		**Neck Lymph Node Status at Last FUV**				**Neck Lymph Node Status at Last FUV**			
		US-N (%)	US-I (%)	US-S (%)	Total (%)	US-N (%)	US-I (%)	US-S (%)	Total (%)
Serum Tg	Tg-N, <0.2 ng/mL	65 (81.3)	0 (0)	1a (1.3)	66 (82.5)	4 (50.0)	0 (0)	0 (0)	4 (50.0)
status	Tg-I, 0.2-0.99 ng/mL	13 (16.3)	0 (0)	0 (0)	13 (16.3)	2 (25.0)	0 (0)	1 a , c , d (12.5)	3 (37.5)
at final FUV	Tg-S, ≥1 ng/mL	1a (1.3)	0 (0)	0 (0)	1 (1.3)	1a (12.5)	0 (0)	0 (0)	1 (12.5)
Total		79 (98.8)	0 (0)	1 (1.3)	80 (100)	7 (87.5)	0 (0)	1 (12.5)	8 (100)

a Details on these patients are reported in Table 3 .

b This patient’s US-I lymph node was not detected at last FUV.

c Data belongs to the same patient.

d This patient had a total follow-up time of five years (FUV-5 and final FUV data are the same).

At the last FUV (mean follow-up time: 6.7 ± 1.2 years), one patient had developed a neck lymph node status classified as US-S, with Tg-N [NPV: 98.8% (95% CI: 93.2% to 100.0%)]. Thirteen patients presented with a Tg-I status and one patient with a Tg-S status ( Table 2 ).

Table 3 presents details on the US-S case and the four patients with Tg-S status. The three patients who presented with a Tg-S status at FUV-5 no longer maintained this status at the last FUV. None of these patients were positive for TgAb at any FUV. At the time of data collection, no additional information was available on the patients with Tg-S or US-S status at the last FUV.

**Table 3:** Characteristics of patients classified as Tg-S or US-I/US-S or both at FUV-5, last FUV or both

Group/Patient No.	Estimated Risk of Recurrence a	RRA	FUV-5			Last FUV		
			Tg and US Status	TSH, mIU/L	TgAb positivity	Tg and US Status	TSH, mIU/L	TgAb positivity
1/1	Low	Y	Tg-S + US-N	3.79	N	Tg-S + US-N	1.02	N
1/2	Low	Y	Tg-S + US-N	3.60	N	Tg-I + US-N	0.29	N
1/3	Low	N	Tg-I + US-N	1.70	N	Tg-S + US-N	1.35	N
1/4	Intermediate	Y	Tg-S + US-N	0.12	N	Tg-N + US-N	0.05	N
1/5	Intermediate	Y	Tg-N + US-N	1.71	N	Tg-N + US-S	1.52	N
2/1	Low	Y	Tg-N + US-I	0.29	N	Tg-N + US-N	10.29	N
2/2	Low	N	Tg-I + US-N	3.28	N	Tg-S + US-N	3.33	N
2/3 b	Intermediate	Y	Tg-I + US-S	0.15	N	Tg-I + US-S	0.15	N

a According to the 2015 ATA Guidelines ( 1 ).

b This patient had a total follow-up time of five years (FUV-5 and last FUV data are the same).

Applying the composite endpoint to define absence of disease (both serum Tg < 1 ng/mL and US-N status) at the last FUV, we obtained an NPV of 97.5% (95% CI: 91.3% to 99.7%).

### Group 2

Table 2 presents the analysis of the data from group 2. At FUV-5, of the eight patients, six had a US-N status. Accordingly, the NPV of the Tg-I + US-N status at FUV-1 was 75.0% (95% CI: 34.9% to 96.8%). One patient with Tg-N had US findings classified as US-I, and another patient with Tg-I had a US-S status. Three patients presented with a Tg-I status and a US-N.

At the last FUV (mean follow-up time: 6.5 ± 1.8 years), the US-I neck lymph node detected at FUV-5 did not persist. The patient with US-S + Tg-I findings had a total follow-up time of five years; therefore, the data reported at FUV-5 and the final FUV belong to the same FUV. Consequently, a total of seven patients had US-N status [NPV of a Tg-I + US-N status at the FUV-1: 87.5% (95% CI: 47.4% to 99.7%)]. At the final FUV, one patient had developed Tg-S status (with US-N) and two other patients presented with Tg-I.

Table 3 presents details on the patient with US-I + Tg-N at FUV-5, the patient with US-S + Tg-I at FUV-5 and the patient with Tg-S + US-N at the last FUV (group 2). None of these patients had positive TgAb. Further details on the patient with Tg-S and the patient with US-S were not available at the time of data collection.

Analysis of the composite endpoint to define absence of disease (serum Tg < 1 ng/mL and US-N status) at the last FUV resulted in an NPV of 75.0% (95% CI: 34.9% to 96.8%).

Table 4 summarizes the NPVs of each group at FUV-5 and the last FUV.

**Table 4:** Negative predictive values by group

	Group 1 (CI)	Group 2 (CI)
FUV-5	100.0% (95.5%-100.0%) a	75.0% (34.9%-96.8%)
Last FUV	98.8% (93.2%-100.0%)	87.5% (47.4%-99.7%)

All NPVs were calculated with a 95% confidence interval (CI).

a One-sided 97.5% CI.

## DISCUSSION

DTC, particularly PTC, is associated with low recurrence and mortality rates ( 5 , 7 , 10 ). Nevertheless, patients’ disease burden is growing due to the increasingly accessible and capable diagnostic procedures, namely the neck US ( 10 , 20 - 22 ). Various studies have already shown that neck USs have a greater likelihood of detecting non-actionable or incorrect sonographic suspicious findings than recurrence of clinically significant structural disease ( 12 - 16 ), which can lead to unnecessary additional interventions for a disease that would have no impact on patients’ quality of life or survival rates ( 11 - 14 ). Current guidelines are assertive regarding the use of Tg, TgAb and TSH measurements for the long-term management of DTC, but they are gradually suggesting that neck USs should be reserved for selected cases and not be performed as a routine exam ( 14 , 18 , 19 ) despite their high sensitivity and ability, in some cases, to detect recurrence before the elevation of Tg levels ( 18 , 23 ).

The main findings of the current study suggest that the follow-up for PTC patients with an excellent response to treatment can be safely based on assessments of unstimulated serum Tg levels without a routine neck US. At the one-year assessment, in patients with US-N, a Tg-N status (excellent response to therapy) – group 1 – strongly predicted the absence of sonographic evidence of cervical lymph node disease at FUV-5 and the last FUV.

Before proceeding with further analysis of the results, it is important to mention one important caveat concerning our data. Due to group 2’s small sample size, its results should be interpreted with caution. This limitation resulted in a wide CI and hence a less precise estimated effect. Consequently, we cannot assertively make similar inferences with indeterminate levels of unstimulated Tg (0.20-0.99 ng/mL); therefore, further discussion of results will focus on the group of patients with an excellent response to treatment (group 1). The reports from the group 1 patients’ neck USs only described one lymph node classified as suspicious (in intermediate-risk patients). Its presence would have been missed if assessment were based on Tg and TgAb alone, as none of these values were suspicious. This finding probably corresponds to false positives; nevertheless, no further data is available, as the report corresponds to this specific patient’s last FUV.

We also reported one patient with a neck lymph node classified as indeterminate at FUV-5. However, we considered it a false positive finding, as the subsequent neck US was normal. This result is consistent with previous studies that showed high rates of false positives from neck USs ( 12 - 14 ) and low probability of detecting clinically relevant structural recurrence ( 12 - 16 , 24 ). Moreover, a recent study by Sek and cols. ( 25 ), conducted on 93 patients with low- and intermediate-risk PTC with a median follow-up time of five years, showed that execution of routine neck USs detected non-actionable findings in 20.4% of patients, which led to unnecessary procedures and interventions.

Our results support evidence from previous observations reported by Grani and cols. ( 17 ) in a study conducted on 226 patients with low- and intermediate-risk PTC. They showed that a one-year serum Tg-N + US-N status was strongly predictive of absence of cervical lymph node disease at the three-year assessment and at the last FUV (NPVs: 98.8% and 98.2%, respectively; median follow-up of 72 months) as well as a one-year Tg-I + US-N status (NPVs: 98.2% at FUV-3 and 94.5% at the last FUV; median follow-up of 86 months). These findings, combined with ours and those reported in previous observations ( 14 , 18 ), further support the hypothesis that subsequent follow-up for low- and intermediate-risk PTC patients with no evidence at the one-year assessment can be safely performed with yearly serum Tg levels and TgAb assessments, without a routine neck US.

Regarding previous neck dissection, we had a high percentage of patients submitted to this procedure (58.8%) due to the less conservative approach to neck dissection in the institutional protocol in force at the time based on the 2009 ATA guidelines. Although we recognize that a previous neck dissection might lead to a higher rate of postsurgical athyroglobulinemia, potentially increasing the sensitivity of thyroglobulin for long-term follow-up ( 26 - 28 ), we believe this finding should not alter the interpretation of our results. Mounting evidence shows that low- and intermediate-risk papillary carcinoma submitted to thyroidectomy without neck dissection can be safely monitored with thyroglobulin ( 1 , 29 - 31 ).

Some limitations of our study must be considered. First, because it is a retrospective and single-center study, the power of the results is limited, as they may not be suitable for other populations. Additionally, there is the constraint associated with the small sample size for group 2 (n = 8), precluding conclusions for unstimulated Tg levels between 0.20 and 0.99 ng/mL. Moreover, the results cannot be extrapolated to patients diagnosed with other, more aggressive variants of DTC, as they exhibit atypical clinical and biological characteristics. Nevertheless, the less aggressive and more classic variants addressed in this study (PTC) represent the vast majority of diagnosed DTC ( 32 - 34 ). The follow-up time may also seem relatively short. However, it comprises the interval in which the vast majority of DTC recurrences are reportedly found, as studies suggest that more than 75% of recurrences occur in the first five years ( 18 , 35 ).

The strengths of our study include the fact that our findings are consistent with those reported by similar studies conducted in different centers. Furthermore, the TGC consists of a team of physicians with extensive experience in the field, who conduct well characterized and extensive routine assessments, which, combined with strict inclusion and exclusion criteria and a rigorous US-report classification (each reviewed and classified by two investigators), allowed for the inclusion of a more uniform sample and reliable data, however leading to a smaller sample size.

In conclusion, our results show that low- and intermediate-risk PTC patients treated with total thyroidectomy with an excellent response to treatment (normal US, undetectable unstimulated Tg and negative TgAb levels) at the 1-year evaluation can be safely monitored with regular unstimulated serum Tg assessments. In these patients, neck USs should be reserved for those patients with rising serum Tg levels and/or TgAb titers.

These results could lead to fewer unnecessary diagnostic procedures and interventions and better quality of life for the patients as well as lower costs in their long-term follow-up. Nevertheless, further prospective studies on this topic should be conducted, and larger samples are necessary to confirm these findings for patients with serum Tg levels between 0.20 and 0.99 ng/mL.
